# The two *α-dox *genes of *Nicotiana attenuata: *overlapping but distinct functions in development and stress responses

**DOI:** 10.1186/1471-2229-10-171

**Published:** 2010-08-11

**Authors:** Anke Steppuhn, Emmanuel Gaquerel, Ian T Baldwin

**Affiliations:** 1 Department of Molecular Ecology, Max-Planck-Institute for Chemical Ecology, Hans-Knöll-Str. 8, Jena 07745, Germany; 2 Molecular Ecology Department/Dahlem Centre of Plant Sciences, Institute for Biology/Free University of Berlin, Haderslebener Str. 9, Berlin 12163, Germany

## Abstract

**Background:**

Plant fatty acid α-dioxygenases (α-DOX) are oxylipin-forming enzymes induced by biotic and abiotic stresses, which also participate in developmental processes. In *Nicotiana attenuata*, herbivory strongly induces the expression of an *α-dox1 *gene. To determine its role, we silenced its expression using *Agrobacterium*-mediated plant transformation with an inverted repeat construct. More than half of the transformed lines showed a severe dwarf growth phenotype that was very similar to the phenotype of tomato plants mutated at a second *α-dox *isoform. This led us to identify the corresponding *α-dox2 *gene in *N. attenuata *and examine the regulation of both *α-dox *genes as well as the consequences of their silencing in plant development and anti-herbivore defense.

**Results:**

The transformed lines exhibiting a dwarf growth phenotype are co-silenced for both *α-dox *genes resulting in a nearly complete suppression of α-DOX activity, which is associated with increases in ABA, JA and anthocyanin levels, all metabolic signatures of oxidative stress. The other lines, only silenced for *α-dox1*, developed similarly to wild-type plants, exhibited a 40% reduction of α-DOX activity resulting in a 50% reduction of its main product *in planta *(2-HOT) and showed no signs of oxidative stress. In contrast to *α-dox1*, the expression of *α-dox2 *gene is not induced by wounding or elicitors in the oral secretions of *Manduca sexta*. Instead, *α-dox2 *is expressed in roots and flowers which lack *α-dox1 *expression, but both genes are equally regulated during leaf maturation. We transiently silenced *α-dox *gene copies with gene-specific constructs using virus induced gene silencing and determined the consequences for plant development and phytohormone and 2-HOT levels. While individual silencing of *α-dox1 *or *α-dox2 *had no effects on plant growth, the co-suppression of both *α-dox *genes decreased plant growth. Plants transiently silenced for both *α-dox *genes had increased constitutive levels of JA and ABA but silencing *α-dox1 *alone resulted in lower *M. sexta*-induced levels of JA, 2-HOT and ABA.

**Conclusions:**

Thus, both *α-dox *isoforms function in the development of *N. attenuata*. In leaf maturation, the two *α-dox *genes have overlapping functions, but only *α-dox2 *is involved in root and flower development and only *α-dox1 *functions in anti-herbivore defense.

## Background

Fatty acid (FA) hydroperoxides are intermediates in different oxylipin pathways controlling plant development [1] and plant responses to stresses [2]. To date, much of the research on oxylipin signals has focused on jasmonic acid (JA) whose biosynthesis starts with the peroxidation of linolenic acid (C18:3) mediated by 13-lipoxygenase (13-LOX) enzymes. In *Nicotiana attenuata*, silencing of *lox3*, a gene coding for a 13-LOX isoform, considerably reduces the accumulation of herbivory-induced jasmonic acid and as a consequence, the accumulation of several direct defense compounds, such as nicotine and trypsin proteinase inhibitors, as well as terpenoid volatiles that function as indirect defenses [3]. Consistent with its important regulatory role in mediating responses to herbivory, *lox3 *expression increases after herbivore attack [4].

In *N. attenuata*, one of the transcripts most strongly elicited by the feeding of different herbivores is an *α-dioxygenase *(*α-dox*), a gene homologue of *Nicotiana tabacum *(Nt) *α-dox *[5-8]. The Nt*α-dox *was first identified as a pathogen inducible oxygenase (PIOX) sharing significant homologies with mammalian prostaglandin endoperoxidases [9]. *α*-DOXs are FA-hydroperoxidases which target the *α*-carbon (C2) of a broad range of FAs [9,10]. *In vitro*, their catalytic activity is characterized by the production of long-chain aldehydes formed from α-hydroperoxy-FA molecules that escape reduction to α-hydroxy-FA and undergo spontaneous decarboxylation [10]. Like LOXs, α-DOXs also use linolenic acid (C18:3) as substrate, but convert it to heptadecatrienal (HDT) *in vitro*. However, quantification of the oxidative products of α-DOX activity in response to bacterial inoculation, revealed that 2-hydroxy-C18:3 (2-HOT) is the major product synthesized in tobacco plants [11]. Because 2-HOT exhibits antimicrobial activity at high concentrations, it may directly protect plants against pathogen attack [11,12]. However, the biological functions of this and other α-DOX products remain largely unknown.

Several lines of evidence indicate that *α-dox *genes are involved in responses to different abiotic and biotic stresses. In tomato roots, for example, ethylene increases *α-dox *transcript accumulation during salt stress [13]. Other abiotic stresses, such as UV-B exposure, heavy metal stress, and cold stress, increase *α-dox *transcript accumulation in *Nicotiana longiflora*, rice and Arabidopsis, respectively [14-16]. However, more work has examined whether α-DOX is involved in a plant's response to pathogens and herbivores. In *Capsicum annuum *transcripts of an α-DOX homolog increase during pathogen infection [17]. In *Arabidopsis thaliana *and *N. tabacum*, the transcriptional up-regulation of *α-dox *is amplified when the infection results in a hypersensitive response [9,18]. Ponce de León et al. [19] showed that *α-dox *gene expression is impaired in salicylic acid (SA)-compromised plants and transgenic *A. thaliana *plants silenced in *α-dox *expression do more rapidly develop severe necrotic lesions in response to incompatible bacteria than do wild-type (WT) plants. This suggests that α-DOX1 activity protects tissues from excessive necrosis; however, the responsible mechanisms remain unknown.

In *N. attenuata*, the effects of pathogen inoculation and treatment with pathogen-derived elicitors on *N. attenuata α-dox *transcript accumulation is much weaker than that of its homologues in other species [5]. The strong transcriptional up-regulation of *N. attenuataα-dox *in response to herbivore attack involves the initial perception of fatty acid amino acid conjugates (FACs), which are herbivore specific elicitors in the oral secretions (OS) of lepidopteran larvae [20]. In addition, the up-regulation of *α-dox *requires the JA-signaling pathway as demonstrated by the lack of *α-dox *transcript accumulation in OS-elicited *lox3*-silenced plants [3]. JA elicitation of *α-dox *transcripts has also been reported in *Oryza sativa *[15] and *N. tabacum *[9]. This regulation suggests an anti-herbivore function for Naα-DOX in *N. attenuata*.

Generally, the increased transcription of *α-dox *genes in plants attacked by herbivores and pathogens suggests a defensive function; however, plant *α*-*dox *transcripts and activity are also regulated during developmental processes. Fatty acidα-oxidation was first reported in peanut seedlings during germination [21]. Also during germination, one of the first α-DOX proteins was isolated in pea. Pea seedlings accumulate Ps*α-dox *transcripts at much higher levels than do leaves and dry seeds [22] and these are lost during maturation [23] and later detected exclusively in roots. Additional evidence for a role in development is suggested by the increasing expression Nt*α-dox *gene during leaf senescence in *N. tabacum *[24].

FA α-oxidation is phylogenetically widespread, as even preparations from green alga form 2-hydroxypalmic acid from palmic acid [25]. The α-DOX proteins identified in different plant species show high amino acid homologies. For example, the amino acid sequence of *N. tabacum *α-DOX shares high amino acid similarities with the proteins in *N. attenuata *(95% identity), *C. annuum *(85% identity), *Solanum lycopersicum *(84% identity), *A. thaliana *(75% identity), and *O. sativa *(63% identity) [18]. In addition to these proteins, referred to as α-DOX1, another α-DOX isoform exists. A second protein, first identified in tomato and *A. thaliana*, shares more homology between species than with the corresponding α-DOX1 sequence of the same species. This second protein, encoded by *α-dox2*, is the *S. lycopersicum feebly *gene, which leads to a dwarf phenotype in a knock-out mutant [26]. The patterns of *α-dox2 *transcript accumulation differ from those of *α-dox1 *in both *S. lycopersicum *and *A. thaliana*. Whereas *α-dox2 *transcript accumulation is not increased by pathogen infection, it is enhanced in seedlings and in wilting leaves 3 to 6 days after detachment [27]. Despite a similar transcriptional regulation of *α-dox2 *in *S. lycopersicum *and *A. thaliana*, its role in plant development appears to be species-specific. Ectopic expression of the At*α-dox2 *in a tomato mutant deficient for *α-dox2 *partially complemented the compromised growth phenotype, but deletion of At*α-dox2 *did not result in a growth phenotype [28]. In summary, although α-DOX proteins are widespread across the plant kingdom their specific functions, presumably in defense and development, remain to be elucidated.

To determine the role of the herbivore responsive *α-dox1 *gene in *N. attenuata*, we used *Agrobacterium *mediated plant transformation with an inverted repeat (IR) construct to silence its expression. More than half of the lines showed a severe dwarf growth phenotype, the other lines developed similarly to WT plants. Though the α-DOX activity was reduced in all lines, it was not detectable in those lines exhibiting a dwarf phenotype. To test whether this resulted from a co-silencing of a second *α-dox *isoform in the dwarf phenotype lines, we used a consensus sequence of *α-dox2 *isoforms in *L. esculentum *and *A. thaliana *to clone a gene fragment of the *N. attenuata α-dox2 *gene. The dwarf phenotype correlated with the co-silencing of the Na*α-dox2 *gene. In contrast to Na*α-dox1*, Na*α-dox2 *transcripts were not induced by wounding or simulations of *Manduca sexta *attack. Instead, *α-dox2 *was more expressed in roots and flowers. To investigate the functions of both isoforms, we transiently silenced both with gene specific constructs using virus induced gene silencing (VIGS) and examined the consequences for plant development and constitutive and induced phytohormone levels.

## Results

### Silencing Na*α-dox *in *N. attenuata *results in two growth phenotypes

**Figure 1:**
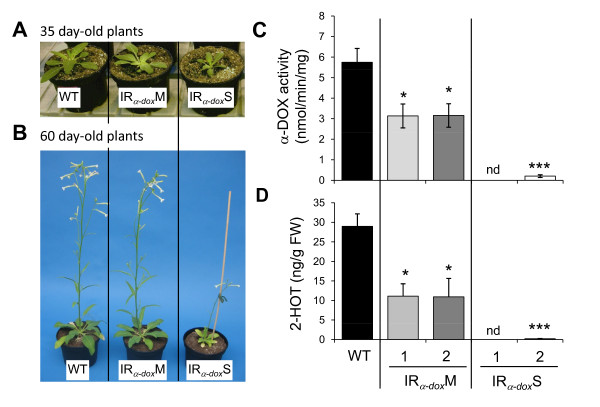
**Silencing Na*α-dox1 *results in lines with two different growth phenotypes correlating with the suppression level of α-DOX activity**. The growth phenotype of **(A) **35-day-old and **(B) **60-day-old WT and T_3 _homozygous *Nicotiana attenuata *plants transformed with an inverted repeat (IR) construct to silence Na*α-dox1*. More than half of 18 independently transformed lines are severely stunted (IR_*α-dox*_S) whereas the other lines (IR_*α-dox*_M) are morphologically indistinguishable from wild-type plants (WT). **(C) **α-DOX activity (mean ± SE of 4 biological replicates) of protein extracts from leaves by *in vitro *measurements of the formation of 8,11,14-heptadecatrienal (HDT). Leaves had been wounded and treated with 2.5 μL of OS_WT _every 30 min for four times. Leaves were harvested 30 min after the last treatment. **(D) **Leaf levels of 2-hydroxyoctadetrienoic acid (2-HOT; mean ± SE of 4 biological replicates), the main α-DOX product *in planta*. Asterisks signify significant differences between IR_*α-dox*_and WT plants (unpaired *t-test *WT vs. lines P < 0.05). nd: not detected.

To examine the function of Na*α-dox1 *[GenBank AF229926], we transformed *N. attenuata *with an IR construct containing a 457 bp fragment of Na*α-dox1 *to silence its expression (Additional file 1). Two distinct growth phenotypes were observed: 11 of the 18 independently transformed lines showed stunted growth (IR_*α-dox*_S), whereas the other lines showed a mild phenotype (IR_*α-dox*_M) and were indistinguishable from WT plants in their growth and development. Plants of the IR_*α-dox*_S lines germinate normally though a higher proportion of infertile seeds were produced by these plants. Young IR_*α-dox*_S seedlings appear similar to WT plants, but already when transplanted into soil two weeks after germination, they are slightly delayed in development. At rosette stage, approximately 35 days after germination, IR_*α-dox*_S plants are clearly delayed with rosette diameters of about a third of that of WT and IR_*α-dox*_M plants (Figure 1A). At flowering, approximately 60 days after germination, IR_*α-dox*_S plants only attained one fourth to a third of the height of WT plants and produced only few flowers (Figure 1B). Whereas WT *N. attenuata *plant in 1 L pots produce between 50 and 120 capsules, homozygous IR_*α-dox*_S plants usually produce between 0 and 5 capsules. The α-DOX activity was reduced in all IR_*α-dox *_lines. However, whereas IR_*α-dox*_M plants showed a reduction of around 40% in α-DOX activity, only very low or no activity was detectable in dwarf IR_*α-dox*_S plants (Figure 1C). The accumulation of the main product of α-DOX *in planta*: 2-HOT was reduced by more than 50% in IR_*α-dox*_M plants compared to WT and was at the detection limit in IR_*α-dox*_S plants (Figure 1D).

### A *α-dox2 *isoform in *N. attenuata *is co-silenced in the IR_*α-dox *_lines

In *S. lycopersicum *and *A. thaliana *a second gene with high sequence similarity to *α-dox1 *was identified [27]. The phenotype of tomato plants mutated at this Sl*α-dox2 *locus (*feebly, divarcata*) is very similar to that of the IR_*α-dox*_S plants [26,28]. A Southern-blot analysis performed by Hermsmeier et al. (2001) has already demonstrated that *N. attenuata*'s genome contains at least two *α-dox *gene copies. We therefore investigated whether a homologue of *S. lycopersicum *and *A. thaliana α-dox2 *may also be present in *N. attenuata *and may have been co-silenced in the IR_*α-dox*_S plants. We used a consensus sequence of *α-dox2 *isoforms in *S. lycopersicum *and *A. thaliana *with low sequence identity to the *N. attenuata α-dox1 *gene to design primers to amplify a putative Na*α-dox2 *gene. We amplified a sequence and cloned it into *Escherichia coli*. Subsequently, a 355 bp gene fragment [GenBank EU681953.1] of the Na*α-dox2 *was sequenced from 3 independent clones. By comparison of this gene fragment to a custom data base of the complete *N. attenuata *transcriptome we identified a cDNA sequence of Na*α-dox2 *of 2290 bp [GenBank HM140643]. The translated amino-acid sequence deduced from this sequence clusters in a phylogenetic tree of plant α-DOX sequences close to the *α-dox2 *isoforms in *S. lycopersicum *and *A. thaliana *but apart from *N. attenuata α-dox1 *(Additional file 2).

**Figure 2:**
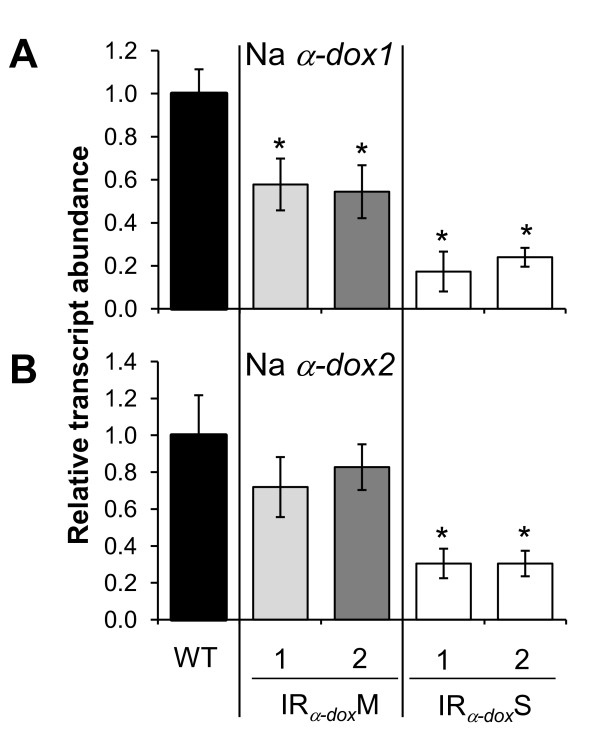
**The stunted growth phenotype of stably transformed IR**_***α-dox***_**S plants is associated with a stronger silencing of Na*α-dox1 *and a co-silencing of Na*α-dox2***. Transcript accumulation (mean ± SE of 5 biological replicates) as measured by SYBR Green-based real time-PCR of (**A**) Na*α-dox1 *and (**B**) Na*α-dox2 *in wild-type *Nicotiana attenuata *(WT) and plants transformed with an inverted repeat (IR) construct to silence Na*α-dox1*. The transcript abundance is expressed relative to WT expression. Whereas lines that are morphologically similar to WT plants (IR_*α-dox*_M) show a moderate silencing of *α-dox1*, severely stunted lines (IR_*α-dox*_S) are more strongly reduced in *α-dox1 *transcripts and co-silenced for the *α-dox2 *gene. Leaves had been wounded and treated with 2.5 μL of OS every 30 min for four times. Leaves were harvested 30 min after the last treatment. Asterisks signify significant differences between IR_*α-dox*_and WT plants (unpaired *t-test *for transcripts of Na*α-dox1*: M1 P = 0.04, M2 P = 0.03, S1 P = 0.001, S2 P = 0.01; of Na*α-dox2*: M1 P = 0.37, M2 P = 0.63, S1 P = 0.01, S2 P = 0.03).

By SYBR Green-based real-time (RT)-PCR with gene specific primers for Na*α-dox1 *and Na*α-dox2 *(for primer specificity see Additional file 3A), transcript accumulation of both genes was determined in IR_*α-dox*_M and IR_*α-dox*_S plants after repeated treatment with *M. sexta *OS to puncture wounds, a treatment known to strongly induce Na*α-dox1 *transcription [29]. Transcript accumulation of Na*α-dox1 *was reduced by 40% in IR_*α-dox*_M and by 85% in IR_*α-dox*_S plants of WT levels (Figure 2A). Moreover, in IR_*α-dox*_S plants transcript accumulation of Na*α-dox2 *transcripts was reduced by 70%, whereas IR_*α-dox*_M plants had Na*α-dox2 *transcript levels comparable to those of WT plants (Figure 2B). Thus, the dwarf phenotype of IR_*α-dox*_S plants is correlated with a concomitant silencing of the Na*α-dox2 *isoform in conjunction with a stronger silencing of the Na*α-dox1 *gene.

### Distinct transcriptional regulation of Na*α-dox1 *and Na*α-dox2*

**Figure 3:**
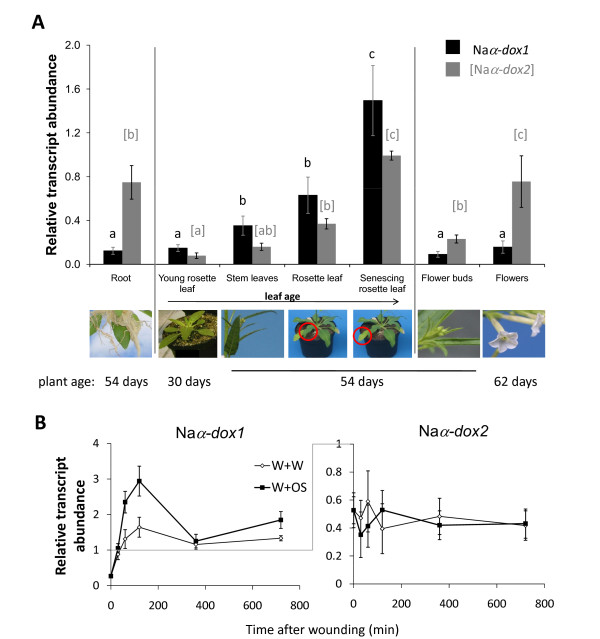
**Transcriptional regulation of Na*α-dox *genes in development and the response to wounding and *Manduca sexta *oral secretions**. (**A**) Mean ± SE (3 biological replicates) of transcript abundance of Na*α-dox1 *and Na*α-dox2 *(relative to *actin *transcripts) in various tissues of developing wild-type *Nicotiana attenuata*. Different letters signify significant differences between different tissues for transcript abundance of Na*α-dox1 *(in black) and Na*α-dox2 *(in gray and brackets) respectively (*ANOVA*s with factor tissue: *α*-*dox1 *F_6,21 _= 18.65, P < 0.0001 and *α*-*dox2 *F_6,21 _= 20.63, P < 0.0001; *Fisher's PLDS *for significant differences P < 0.036). Paired *t-tests *were used to test for significant differences between transcript abundance of Na*α-dox1 *and Na*α-dox2 *in the tissues (roots: P = 0.005, young rosette leaves P = 0.047, stem leaves P = 0.053, old rosette leaves P = 0.047, senescing leaves P = 0.075, flower buds P = 0.02, flowers P = 0.03). (**B**) Mean ± SE (5 biological replicates) of transcript abundance of Na*α-dox1 *and Na*α-dox2 *(relative to *actin *transcripts) in leaves from *N. attenuata *rosette stage plants wounded at the first fully expanded source leaf with a fabric pattern wheel every 30 min for four times. To the resulting puncture wounds 2.5 μL of either water (W) or *M. sexta*'s oral secretions (OS) were added. Only the expression of Na*α-dox1 *but not that of Na*α-dox2 *increased after wounding, which was amplified by OS application (2 factorial *ANOVA *with factors treatment and time: Na*α-dox1 *P < 0.0046 for both factors the interaction; for Na*α-dox2: *P > 0.45 for both factors the interaction).

To evaluate the roles that Na*α-dox1 *and Na*α-dox2 *play during development we analyzed transcripts of both genes in various tissues of developing *N. attenuata *plants. Both genes show strong tissue specific differences in their transcript abundance (Figure 3A). In *N. attenuata *leaves, transcripts of both Naα-*dox *genes accumulate with plant and leaf maturation resulting in highest transcript levels in senescing leaves. In leaf tissues, Na*α-dox1 *transcripts are generally more abundant than Na*α-dox2 *transcripts. Though this difference was only significant in the young rosette leaves, the same tendency was observed in stem leaves, rosette leaves (paired *t-test *P = 0.053), and senescing leaves. The difference between the two Na*α-dox *genes was more pronounced in roots, flower buds, and flowers, but in these tissues Na*α-dox2 *transcripts were much more abundant than those of Na*α-dox1*. In brief, Na*α-dox2 *expression is increased in roots, during flower formation, as well as during leaf maturation and senescence, but only the latter process also enhanced Na*α-dox1 *transcripts.

As both Na*α-dox *genes have a similar transcriptional regulation during normal leaf maturation, the question arises, whether the Na*α-dox2 *is also regulated during herbivore attack as is known for the Na*α-dox1 *gene in the leaves of *N. attenuata*. Therefore, we compared the inducibility of Na*α-dox1 *and Na*α-dox2 *transcript accumulation by wounding and herbivore specific elicitors. By SYBR Green-based RT-PCR gene specific transcript accumulation of Na*α-dox1 *and Na*α-dox2 *genes was determined in WT *N. attenuata *plants after repeated treatment with *M. sexta *OS to puncture wounds. As known from previous studies [5,30] transcript accumulation of Na*α-dox1 *is increased significantly by wounding and is further up-regulated by the treatment of the wounds with *M. sexta *OS (Figure 3B). Interestingly, the Na*α-dox2 *gene does not respond to wounding or to the application of *M. sexta *OS.

### Co-silencing of both Na*α-dox *genes increases constitutive levels of ABA, JA and anthocyanins

We measured phytohormone levels in leaves to characterize the metabolic consequences of silencing either Na*α-dox1 *or additionally Na*α-dox2*. We quantified levels of JA, abscisic acid (ABA) and SA because previous studies indicated that α-DOX is involved in the physiological processes mediated by these phytohormones. The major signaling molecule in herbivore induced responses, JA, has been shown to be required for *α-dox1 *expression in *N. attenuata *[3] and levels of ABA and SA have been reported to regulate *α-dox *expression in Arabidopsis and tomato plants [9,13]. The IR_*α-dox*_S plants that were co-silenced for both Na*α-dox *isoforms showed strongly increased levels of the phytohormone ABA, but the IR_*α-dox*_M plants did not (Additional file 4A). In 39-days old rosette stage plants, we found an almost 3-fold increase in constitutive ABA levels and a slight increase in constitutive JA levels (Additional file 4B) in IR_*α-dox*_S plants compared to WT. Similarly, constitutive levels of SA were increased in IR_*α-dox*_S plants compared to WT (Additional file 4C).

Because the retarded growth phenotype of the IR_*α-dox*_S plants is already evident at the seedling stage, we also measured anthocyanins in the seedling stage. Anthocyanins are general indicators of stress responses and thought to be involved in a stress signaling response via reactive oxygen species (ROS) [31]. In seedlings of an IR_*α-dox*_S line, levels of total anthocyanins were more than five-fold higher than WT levels 11 and 15 days after germination started (Additional file 3B). Seedlings of a second IR_*α-dox*_S line had anthocyanin levels increased by more than 10% 19 days after sowing. The increase in anthocyanins in IR_*α-dox*_S lines compared to WT seedlings was significant (Mann-Whitney *U-test*s P < 0.05 for 11 and 15 days old seedlings of both lines and for 19 days old seedling of one line).

### Consequences of silencing Na*α-dox1 *and Na*α-dox2 *individually and concomitantly

**Figure 4:**
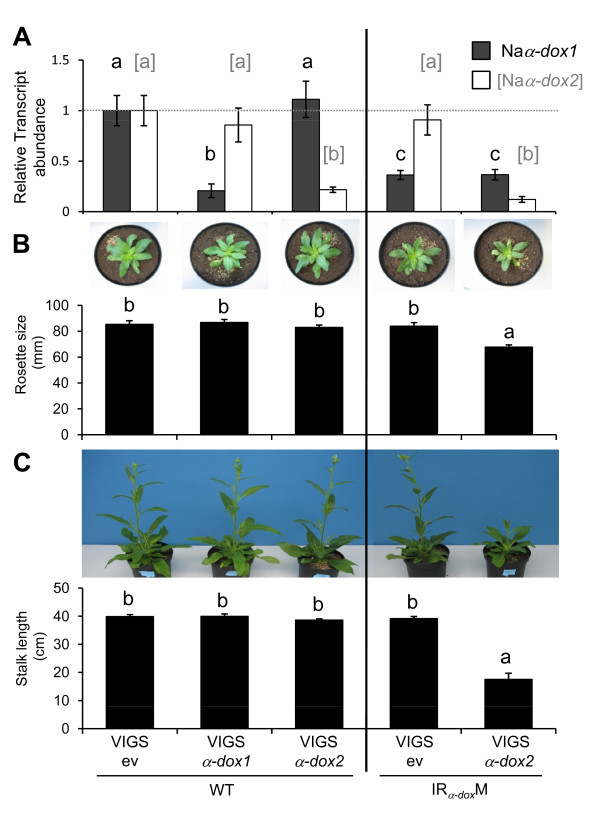
**Co-silencing of Na*α-dox1 *and Na*α-dox2 *reduced plant growth of *Nicotiana attenuata***. (**A**) Relative transcript abundance of Na*α-dox *genes (mean ± SE of 5 biological replicates) in plants subjected to virus-induced gene silencing (VIGS). Wild-type plants (WT) and plants stably silenced for Na*α-dox1 *using an inverted repeat (IR) construct were inoculated with VIGS constructs when 22 days old. Gene expression levels relative to empty vector controls (ev) confirm the gene specific silencing of Na*α-dox1 *and Na*α-dox2 *and was analyzed in rosette leaves by SYBR Green-based real time PCR (**B**) Rosette diameter of 39-days old and (**C**) stalk length of 60-days old plants (mean ± SE of 11 biological replicates) silenced for Na*α-dox1 *and Na*α-dox2 *by VIGS. Stable and transient silencing of only Na*α-dox1 *as well as transient silencing of only Na*α-dox2 *did not affect plant growth, but the silencing of Na*α-dox2 *in a stable line silenced for Na*α-dox1 *resulted in stunted growth. Different letters signify significant differences in growth parameters or transcript abundance of Na*α-dox1 *(in black) and Na*α-dox2 *(in gray and brackets) respectively (*ANOVA*s with factor transformant: for rosette diameters F_4,50 _= 12.81, P < 0.0001 for stem length F_4,50 _= 197.87, P < 0.0001; *Fischer's PLSD *comparisons between VIGS*α-dox2 *in IR_*α-dox*_M vs. all other transformants P < 0.0001, whereas for comparisons between all other transformants P > 0.21).

To disentangle the single and combined effects of Na*α-dox1 *and Na*α-dox2 *silencing we created gene-specific silencing constructs and transiently silenced Na*α-dox1 *and Na*α-dox2 *separately and in combination using VIGS and examined growth parameter. That VIGS constructs were gene specific was confirmed by real time PCR for Na*α-dox1 *and Na*α-dox2 *genes in VIGS plants (Figure 4A). To compare VIGS of Na*α-dox1 *with stably transformed in IR_*α-dox*_M lines and to IR_*α-dox*_S lines we inoculated in addition to WT also IR_*α-dox*_M plants with the empty vector (ev) and the VIGS construct to specifically silence Na*α-dox2*. Stable and transient silencing of only Na*α-dox1 *as well as transient silencing of only Na*α-dox2 *did not affect plant growth, but the additional silencing of Na*α-dox2 *in a stable line silenced for Na*α-dox1 *resulted in stunted growth (Figure 4B, C). Thus, VIGS*α-dox2 *in IR_*α-dox*_M transformants resembled the growth phenotype when both genes were co-silenced in stably transformed IR_*α-dox*_S lines.

**Figure 5:**
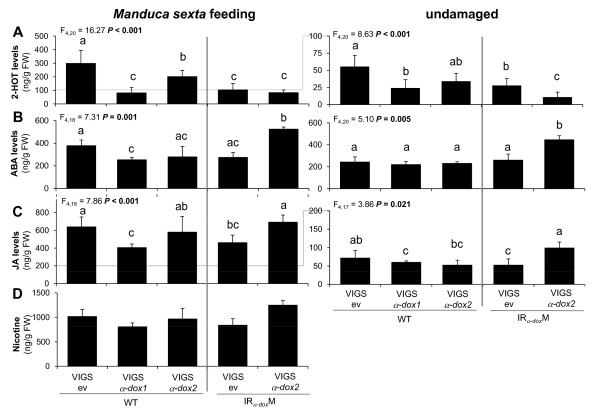
**Silencing Na*α-dox1 *and/or Na*α-dox2 *in *Nicotiana attenuata *differentially affects defense 2-HOT, hormone and nicotine accumulation**. Mean ± SE (*n *= 6 induced & 5 constitutive plants) of leaf levels of (**A**) 2-HOT, (**B**) ABA, (**C**) A, and (**D**) nicotine of plants that had been fed on by *Manduca sexta *larvae for 4 days or left undamaged. Plants were transiently silenced for Na*α-dox1 *and Na*α-dox2 *by virus-induced gene silencing (VIGS) or transformed with an empty vector (ev). Wild-type plants (WT) and plants stably silenced for Na*α-dox1 *using an inverted repeat (IR) construct were inoculated with VIGS constructs when 22 days old. Larvae were applied on day 39. Two-factorial *ANOVA*s with factor transformant and factor *M. sexta *feeding revealed significant effects of (2-HOT levels: transformant F_4,40 _= 23.27, *M. sexta *F_1,40 _= 203.41, interaction F_4,40 _= 5.07, all P < 0.002; ABA levels: transformant F_4,35 _= 10.53, P < 0.001, *M. sexta *F_1,35 _= 8.08, P = 0.007, interaction F_4,35 _= 0.82, P = 0.52; JA levels: transformant F_4,35 _= 10.15, P < 0.0001, *M. sexta *F_1,35 _= 1058.2, P < 0.0001, interaction F_4,35 _= 0.41, P = 0. 80). Different letters signify significant differences according to 1-factorial *ANOVA*s and *Fisher's PLDS *at P < 0.05 (*ANOVA *P and F values are given in the graph).

To investigate the role of Na*α-dox *genes in the plant response to herbivory, we analyzed phytohormones regulating physiological processes that α-DOX has been associated with and induced nicotine accumulation in the plants transiently silenced for either Na*α-dox1 *or Na*α-dox2 *or both. Further, we accessed the contribution of Na*α-dox1 *and Na*α-dox2 *to constitutive and *M. sexta*-induced 2-HOT production. Constitutive 2-HOT levels were significantly reduced in plants silenced for Na*α-dox1 *or Na*α-dox2 *(Figure 5A) with the strongest reduction in plants silenced for both genes. Herbivory by *M. sexta *significantly increased 2-HOT levels. This induction was compromised in all plants silenced in Na*α-dox1 *(*Fisher's PLSD *VIGSev in WT vs. *α-dox1 *silenced transformants P < 0.001) but silencing only Na*α-dox2 *accounted for a minor reduction in herbivory-induced 2-HOT (*Fisher's PLSD *VIGS*α-dox2 *in WT vs. all transformants silenced for Na*α-dox1 *P < 0.004).

The key hormone for responses during pathogen infection salicylic acid (SA) was not significantly affected by the feeding of *M. sexta *but by the expression of α-DOX in the transformed lines (Additional file 4D, E). SA tended to be reduced in all α-*dox *silenced plants with and without herbivory by *M. sexta*, however, this trend was only significant for the comparison between WT plants inoculated with VIGSev and VIGS*α-dox2*.

Feeding by *M. sexta *significantly increased ABA levels (Figure 5B). Constitutive and *M. sexta*-induced levels of ABA are significantly enhanced in plants silenced for both Na*α-dox *genes (*Fisher's PLSD *VIGS*α-dox2 *in IR_*α-dox*_M vs. all other transformants P < 0.001). Silencing Na*α-dox1 *decreases *M. sexta*-induced ABA levels, as ABA levels in VIGS*α-dox1 *plants were significantly lower compared to VIGSev WT plants and the same tendency was observed in VIGSev in IR_*α-dox*_M plants (*Fisher's PLSD *VIGSev in WT vs. VIGSev in IR_*α-dox*_M P = 0.06). As expected, JA levels were strongly increased in response to *M. sexta *feeding (Figure 5C). The levels of induced JA were lower in plants transiently and stably silenced only for Na*α-dox1 *compared to VIGSev WT (*Fisher's PLSD *VIGSev vs. VIGS*α-dox1 *P = 0.0007, VIGSev in WT vs. VIGSev in IR_*α-dox*_M P = 0.007), but not in those silenced for *α-dox2 *(*Fisher's PLSD *VIGSev vs. VIGS*α-dox2 *P = 0.32). Plants silenced for both Na*α-dox *genes had higher levels of JA compared to plants silenced for Na*α-dox1 *alone, which was already apparent in constitutive JA levels (*Fisher's PLSD *VIGS*α-dox2 *in IR_*α-dox*_M vs. other transformants P < 0.03 except vs. VIGSev WT P = 0.70) and did not result from an increased induction after *M. sexta *feeding. Induced nicotine accumulation in VIGS plants corresponded well with the observed JA induction pattern and was lower in the plants only silenced for Na*α-dox1*, but not in those silenced for Na*α-dox2 *(Figure 5D). However, the data with heterogeneous variances were not normally distributed and no transformation for the nicotine data allowed for parametric statistical analysis; the Mann-Whitney *U-test *only detected a significant difference between plants silenced for Na*α-dox1 *and those silenced for both Na*α-dox *genes (VIGS*α-dox1 *WT vs. VIGS*α-dox2 *in IR_*α-dox*_M P = 0.01) because nicotine levels were highest in the latter plants.

These results indicate that the effects of individual and simultaneous silencing of the two Na*α-dox *genes exhibit opposing effects likely resulting from different causes. Whereas stable and transient silencing of the Na*α-dox1 *gene weakens the herbivore-induced plant response with respect to JA and ABA as well as secondary metabolite production, the silencing of both Na*α-dox *genes, which stunts growth, results in higher constitutive levels of diverse stress-related traits, some of which are likely to also be elicited by herbivore attack.

## Discussion

The function of the α-DOX pathway, which is widespread in the plant kingdom, is poorly understood. Here we identify the Na*α-dox2 *gene in *N. attenuata *and demonstrate that α-oxidation of fatty acids plays an important role during its development and that Na*α-dox1 *and Na*α-dox2 *differ in their transcriptional regulation. Both genes are transcriptionally up-regulated during leaf maturation and senescence and silencing both genes results in retarded growth of *N. attenuata *plants. However, the distinct expression patterns of the two Na*α-dox *genes indicate a more important role of the Na*α-dox2 *gene during plant development, especially in root and flower development and seedling maturation. The strong transcriptional regulation of Na*α*-*dox1 *in response to herbivory and the reduced induction of JA when Na*α-dox1 *is silenced suggest a role in plant defense responses, whereas Na*α-dox2 *shows no transcriptional regulation in response to herbivory and its silencing did not change the elicitation of JA. We infer from the different patterns of transcriptional regulation that the two Na*α-dox *genes contribute differently to functions in primary and secondary plant metabolism in *N. attenuata*.

### Functional diversification of plant *α-dox *genes

We identified the Na*α-dox2 *gene and obtained a 2290 bp cDNA sequence from a full transcriptome data base of *N. attenuata*. The Na*α-dox2 *likely corresponds to an orthologue of Arabidopsis and tomato*α-dox *genes with which they share 89% and 79% sequence homology, whereas sequence homology between Na*α-dox1 *and Na*α-dox2 *is 69%. The duplication of *α-dox *genes likely occurred early in the evolution of eudicots before the split of Solanaceae and Brassicaceae, since the orthologues between species contain significantly more homologies than do the paralogues within species.

Stable transformation of *N. attenuata *plants with a silencing construct for Na*α-dox1 *resulted in two distinct growth phenotypes. Whereas growth and development of IR_*α-dox*_M lines with a ca. 40% reduction of α-DOX activity were indistinguishable from those of WT plants, about 60% of the generated lines with low or undetectable α-DOX activity were highly stunted (IR_*α-dox*_S). Stable transformations were performed before the *α-dox2 *paralogues were known and gene specific real time PCR revealed that these two phenotypes correlated with a dissimilar silencing of Na*α-dox1 *and Na*α-dox2 *in the respective lines. Whereas IR_*α-dox*_M plants are reduced in Na*α-dox1 *transcript accumulation by 40%, they are not significantly altered in Na*α-dox2 *transcript accumulation. IR_*α-dox*_S lines, on the other hand, are reduced by 85% in Na*α-dox1 *and 70% in Na*α-dox2 *transcript accumulation. The Na*α-dox1 *silencing construct shares 72% sequence similarity with the Na*α-dox2 *gene and contains a 24 nucleotide match with only one mismatch, which was probably sufficient for the co-silencing of both Na*α-dox *genes in the majority of transformed lines. The stunted growth could either result from a concomitant silencing of both Na*α-dox *genes or the loss of the Na*α-dox2 *function alone. The latter hypothesis is consistent with the stunted growth phenotype of *feebly *[26] and *divaricata *mutants [32] in tomato, which result from insertional mutagenesis at the locus of the Sl*α-dox2 *homologue. However, *Arabidopsis *mutants of At*α-dox2 *do not show any developmental abnormalities, indicating that the developmental role of *α-dox2 *may be restricted to a specific clade e.g. Solanaceae. Alternatively, *α-dox *functions in *N. attenuata *may also be distinct from those in tomato, and the stunted growth of in IR_*α-dox*_S lines is the consequence of an almost complete loss of total α-DOX activity due to a silencing of both Na*α-dox *genes.

### Silencing both Na*α-dox *genes is required to stunt plant growth

By using VIGS-constructs specific for Na*α-dox1 *and Na*α-dox2 *we recognized that silencing Na*α-dox1 *and Na*α-dox2 *individually did not affect growth, but silencing both genes concomitantly decreased plant growth. This finding is consistent with the hypothesis that the stunted growth phenotype results from a reduction in total α-DOX activity and indicates that the α-oxidation activity of the enzymes of both genes can compensate for the loss of function of the other. With *in vitro *assays, Bannenberg et al. [28] demonstrated that tomato Sl*α-dox1 *and Sl*α-dox2 *have a similar range of fatty acid substrates and activities. In *N. attenuata *both Na*α-dox *genes can function in plant development, whereas in tomato the loss of Sl*α-dox2 *function is sufficient to stunt growth and in Arabidopsis the loss of both At*α-dox *genes does not affect growth [28]. The normal growth of *N. attenuata *plants silenced for Na*α-dox2 *by VIGS may alternatively be explained by a lower silencing efficiency compared to the Sl*α-dox2 *tomato insertional mutant, however this seems unlikely given that VIGS reduced Na*α-dox2 *transcripts by 80% and silencing by stable transformation only reduced transcript levels by 70% in the dwarf IR_*α-dox*_S plants.

That the stunted growth in stably transformed IR_*α-dox*_S lines is more pronounced than in the VIGS plants co-silenced for Na*α-dox1 *and Na*α-dox2 *is probably due to greater effects of silencing a gene early than later in development. The growth of IR_*α-dox*_S plants was already delayed two weeks after germination. Though we did not observe obvious growth differences between WT and IR_*α-dox*_S seedlings, the importance of α-DOX activity during seedling maturation in *N. attenuata *is supported by highly increased levels of anthocyanins in IR_*α-dox*_S seedlings. Increased levels of anthocyanins are also described for tomato mutants lacking Sl*α-dox2 *function [28]. The importance of α-oxidation of fatty acids during germination processes is also suggested by the increased transcript accumulation and enzymatic activity of a Ps*α-dox *during the first days of germinating pea plants [23]. Increased levels of anthocyanins and ABA are often associated with oxidative stress [33] and levels of ABA are also increased in IR_*α-dox*_S plants. Additionally, constitutive levels of the phytohormones JA and SA are increased in IR_*α-dox*_S plants. Thus, strong silencing of both Na*α-dox *genes in IR_*α-dox*_S plants could have led to severe physiological stress resulting in increased levels of several stress-related metabolites such as several phytohormones. Alternatively, higher levels of constitutive phytohormones could be a consequence of less cellular expansion in these lines, rather than specific effects on specific metabolic pathways. None of these changes were observed in IR_*α-dox*_M plants indicating that a moderate silencing of Na*α-dox1 *does not result in comparable physiological stress during development.

### Both Na*α-dox *genes have overlapping but distinct roles in plant development

Transcript accumulation of Na*α-dox1 *and Na*α-dox2 *in various tissues of developing *N. attenuata *plants were determined to characterize their endogenous roles. In roots, transcript accumulation of Na*α-dox2 *is very high, whereas it is very low for Na*α-dox1*, which is consistent with previous studies in *N. attenuata *[5] that found no Na*α-dox1 *expression in roots. The root specific expression of Na*α-dox2 *distinguishes *N. attenuata *from pea, Arabidopsis and tomato, in which *α-dox1 *is expressed in roots but not *α-dox2*. Bannenberg et al. [28] found no detectable At*α-dox2 *transcripts in roots of Arabidopsis and only very low levels of Sl*α-dox2 *transcripts but high levels of one Sl*α-dox1 *isoform in tomato roots. In Arabidopsis, the At*α-dox1 *promoter is exclusively active in roots whereas the At*α-dox2 *promoter is exclusively active in shoots [19,28,34]. In pea seedlings, Ps*α-dox *is almost exclusively expressed in roots and this gene shares a greater degree of amino acid sequence similarity with Nt*α-dox1 *and At*α-dox1 *than with At*α-dox2 *[23]. In this regard, the stunted phenotype of IR_*α-dox*_S lines could also result from silencing Na*α-dox2*, which may have interfered with root development causing the observed growth and stress responses. IR_*α-dox*_S lines are characterized by a small main root and very few lateral roots; to determine whether this is a cause or a consequence of the dwarf growth, grafting experiments would be required. However, the loss of function of Na*α-dox2 *in root development can only contribute to but not cause the dwarf phenotype, as the silencing of both Na*α-dox *genes is required to stunt growth. In *N. attenuata*, the transcripts of both Na*α-dox *genes accumulate during leaf maturation and natural senescence with levels of Na*α-dox1 *exceeding those of Na*α-dox2*. This is in contrast to tomato and Arabidopsis, in which At*α-dox2 *and Sl*α-dox2 *transcripts only accumulate in response to the artificial senescence stimulus of leaf detachment [27,28], whereas neither *α-dox1 *nor *α-dox2 *genes accumulate in leaves attached to plants. Moreover, in contrast to *N. attenuata*, in which the levels of Na*α-dox1 *transcripts exceed those of Na*α-dox2*, in Arabidopsis and tomato At*α-dox1 *and Sl*α-dox1 *transcripts are barely detected in leaves and showed only an extremely weak response to leaf detachment. However, the results in *N. attenuata *are in line with the increasing expression of Na*α-dox1 *gene during natural leaf senescence in *N. tabacum *[24]. We propose that in *Nicotiana *species *α-dox *genes may be involved in the regulation of senescence, whereas in Arabidopsis and tomato increasing oxidative stress in the dying tissue of a detached leaf may be at work. The loss of function of both Na*α-dox *genes during leaf maturation may be involved in the dwarf phenotype of the IR_*α-dox*_S lines as the overlapping transcriptional regulation of Na*α-dox1 *and Na*α-dox2 *in leaves corresponds well to the observation that silencing of both genes is necessary to stunt growth.

During flower maturation in *N. attenuata*, only Na*α-dox2 *transcripts accumulate, so that they are twofold higher in mature flowers than in flower buds. Consistent with Hermsmeier et al. [5], who found no transcripts of Na*α-dox1 *in flowers of *N. attenuata*, we found very low transcript abundance of Na*α-dox1 *in flowers. However, in Arabidopsis, promoters of both At*α-dox *genes are active in anthers and the At*α-dox2 *promoter was additionally shown to be active in the ovaries and siliques [19,28]. In distylous species of *Turnera*, a homologue of *α-dox1 *is specifically expressed in short-styled plants [35]. Interestingly, long chain aldehydes, similar to those generated *in vitro *by the activity of α-DOX proteins, are emitted by many flowers [36]; however, the role of *α-dox *genes during flower maturation remains unknown and is likely not involved in causing the stunted phenotype.

### Only Na*α-dox1 *is involved in responses to herbivory

The similar regulation of the two Na*α-dox *genes in *N. attenuata *leaves is restricted to developmental regulation, however in contrast to Na*α-dox1*, the Na*α-dox2 *gene does not respond to wounding or the elicitors in the OS of *M. sexta *larvae. In agreement with its up-regulation during herbivory, Na*α-dox1 *contributed most to the herbivore-inducible 2-HOT level. Silencing of Na*α-dox1 *by VIGS or stable transformation reduced herbivore-inducible 2-HOT levels by 60% while silencing only Na*α-dox2 *had only a marginal effect. Accordingly, we found no effect of silencing Na*α-dox2 *by VIGS on induced responses to *M. sexta *herbivory, whereas stable and transient silencing of Na*α-dox1 *decreased the *M. sexta*-induced levels of JA by 30% and tended to decrease induced levels of the direct defense metabolite, nicotine. VIGS of Na*α-dox1 *also reduced *M. sexta*-induced ABA levels by 25%. Thus, Na*α-dox1 *plays an important role in the regulation of the plant's response to herbivory, whereas silencing Na*α-dox2 *did not affect induced phytohormone levels. VIGS of both Na*α-dox *genes concomitantly only increased constitutive levels of JA and ABA and strongly decreased constitutive levels of 2-HOT which is consistent with the phytohormone phenotype in stably transformed plants co-silenced for Na*α-dox1 *and Na*α-dox2*. Because plants silenced for both Na*α-dox *genes display the loss of function in development and in the plant's response to herbivory, the specific role of Na*α-dox1 *during herbivory should be further investigated using IR_*α-dox*_M plants and plants silenced by VIGS for Na*α-dox1*. A kinetic analysis of JA induction in the Na*α-dox1 *silenced plants may reveal potential feedback regulation by 2-HOT, as we show here that Na*α-dox1 *gene expression is mediated by but also affects JA signaling.

## Conclusions

In this study we identified the *α-dox2 *isoform in *N. attenuata *and revealed that both Na*α-dox *genes function in development but that only Na*α-dox1 *functions in anti-herbivore defense. The conditions that regulate the two *α-dox *isoforms and the consequences of their loss of function in *N. attenuata *and other plants share commonalities and clear differences supporting quite divergent functions in different plant species. Interestingly, phylogenetic relation is a poor predictor of the regulation of *α-dox *genes in different plant species (see Additional file 5 for a summary of the putative functions in a phylogenetic context), but transcriptional regulation is suggested to determine function of *α-dox *genes, because the enzymatic activity is largely similar for the *α-dox *paralogues within species as well as for the orthologes among species. This suggests that the α*-*DOX pathway is a relatively plastic metabolic route in plant primary metabolism which became repeatedly co-opted for specialized functions associated with adaptation to environmental stress. A better mechanistic understanding of the role of *α-dox *genes during plant development and stress responses will help to explain the differences and seemingly convergent similarities of *α-dox *regulation in different plant species.

## Methods

### Plant material & transformation

We used inbred lines of *Nicotiana attenuata *Torr. *ex *Watson derived from field-collected seeds (Baldwin, 1998) for transformation and all experiments. Seed germination and *Agrobacterium tumefaciens *(strain LBA 4404)-mediated transformation procedure are described in Krügel et al. [37]. All plants were grown in the glasshouse in 1 L pots at 26-28°C under 16 h of light supplied by Philips Sun-T Agro 400- or 600-W sodium lights.

To silence the expression of the *N. attenuata α-dox1 *gene [GenBank AF229926], a 458 bp fragment [5] was cloned twice in an inverted-repeat orientation into a pRESC5 transformation vector. T1 plants were screened for resistance to hygromycin (*hygromycin phosphotransferase II gene *from pCAMBIA-1301 [GenBank AF234297] contained in the pRESC5 vector). Homozygosity was determined by resistance screening of the T2 plants. Flow cytometric analysis described by Bubner et al. [38] revealed that all the lines were diploid. Each transformed line harbored only a single insertion, as determined by Southern blot analysis (Additional file 3C) performed on EcoRV-digested genomic DNA with a probe specific for the selective marker gene *hygromycin phosphotransferaseII *(plasmids used for transformation were included as a positive control and additionally digested with XhoI). For the experiments we used two IR_*α-dox*_S lines (line 1: A03-77; line 2: A03-137) with stunted and two IR_*α-dox*_M lines with normal growth (line 1: A03-282; line 2: A03-75), whereas IR_*α-dox*_M line 2 was used in the VIGS experiment.

For virus induced gene silencing, PCR amplified fragments of Na*α-dox1 *and Na*α-dox2 *(each fragment was 187 bp long, for specific sequence see Additional file 1) were digested with BamHI and SalI and each cloned into the pTV00 vector digested with the same restriction enzymes. The pTV00 vector is a 5.5-kb plasmid with an origin of replication for *Escherichia coli *and *A. tumefaciens *and a gene for kanamycin resistance (Ratcliff et al., 2001). Early rosette-stage plants (3 weeks old) were pressure-injected with *A. tumefaciens *(strain GV3101) harboring either the pTV*α-dox1*, pTV*α-dox2 *or the empty vector (ev) control pTV00. A *phytoene desaturase*-silencing construct (pTV*pds*) that causes photobleaching was used as a visible positive control for the VIGS process in all experiments. Inoculation and constructs are described in Saedler and Baldwin [39]. When the leaves of *phytoene desaturase-*silenced plants began to bleach (17 days after inoculation) leaves of *α-dox*-silenced and ev-inoculated plants were used.

### Plant treatments

Experiments with stable transformants were performed with T_3 _generation IR_*α-dox *_plants. Analysis of induced responses (phytohormones, α-DOX activity, and transcriptional changes) was performed with four to five week-old (rosette-stage) plants and treatments were randomly assigned. To mimic herbivory, the first fully expanded source leaf was wounded four times with a fabric pattern wheel with a row of puncture wounds on each side of the midrib and each row received 5 μL of a 1:1 *v/v *dilution of the oral secretions of *M. sexta*. At specified time points, leaves were excised, immediately frozen in liquid nitrogen, and stored at -80°C until use.

Na*α-dox1 *and Na*α-dox2 *constitutive transcript abundances were measured in different tissue parts from WT *N. attenuata *plants. The entire young rosette-stage plant was harvested 30 days after germination. Roots, green or senescing yellow rosette leaves, stem leaves (S5-S8) and flower buds were harvested from 54-day old plants. Complete flowers were harvested 62 days after germination.

Plants that had been subject to virus-induced gene silencing were measured for rosette diameter 17 days after inoculation (at which time there were no differences in plant size) and for stalk elongation 38 days after inoculation. Half of the plants received a *M. sexta *neonate on the transition leaf after inoculation and these larvae were allowed to feed for 4 days. On day 4, leaves on which neonates were feeding were harvested for metabolite measurements. Eggs of *M. sexta *were from North Carolina State University (Raleigh, NC, USA) and maintained in a growth chamber (Snijders Scientific, Tilburg, Netherlands) at 26°C 16 h light, 24°C 8 h darkness until the larvae hatched.

### Identification of the Na*α-dox2 *gene

Primers were designed according to a consensus sequence of *α-dox2 *isoforms in *S. lycopersicum *and *A. thaliana *which have a low sequence identity to the Na*α-dox1 *gene. The resulting PCR amplicon was cloned into pGEM-T Easy plasmid (Promega), transformed into *Escherichia coli *competent cells (JM109) and subsequently sequenced on a Genetic Analyzer 3100 (Applied Biosystems, Darmstadt, Germany) from 3 independent clones. The resulting 355 bp gene fragment of the Na*α-dox2 *was compared to a data base of the complete transcriptome of *N. attenuata *(VERTIS Biotechnologie AG, Germany) and a 2290 bp cDNA sequence of the Na*α-dox2 *gene was identified.

### Analysis of transcript expression by quantitative real time PCR

Total RNA was extracted with trizol following the TIGR protocol available under http://www.jcvi.org/potato/sol_ma_protocols.shtml and cDNA was synthesized from 150 ng RNA using MultiScribe™ reverse transcriptase (Applied Biosystems, Darmstadt, Germany). Quantitative real time PCR (ABI PRISM™ 7000; Applied Biosystems) was conducted using the quantitative PCR™ core reagent kit (Eurogentec, Seraing, Belgium) and gene specific primers for SYBR Green-based quantitative PCR (Na*α-dox1 *forward primer: GTGTTGCTAGGTACAATGAA TTTC; Na*α-dox1 *reverse primer: CAACCATCAGATCCAATTCTTCT; Na*α-dox2 *forward primer: GTTCCACGGTACAACGAGTTCA; Na*α-dox2 *reverse primer: AACTTGGAGATCTAGCTTCTCA). PCR conditions were used according to the manufacturer's recommendations. For each treatment/line combination cDNA of 3 biological replicates were repeated with 3 technical replicates. Relative expression levels were calculated according to a linear standard curve of the threshold cycle number regressed against the log concentration of a dilution series of cDNA standards that were analyzed on each 96-well plate. The expression of *actin *[GenBank EU273278.1] was used as an internal reference to normalize cDNA concentrations.

### Analysis of α-DOX activity

Leaves (pooled from 5 plants) were crushed in liquid nitrogen and 250 μL extraction buffer (0.1 M Tris-HCl pH 7.5) were used for every 100 mg tissue. Leaf tissues were subsequently completely homogenized by being vortexed at 4°C for 45 min. After centrifugation, protein concentration of the resulting supernatants was measured using a Bio-Rad protein assay kit with bovine serum albumin (BSA) as a standard. The α-DOX activity was assayed indirectly by measuring HDT formation in response to α-linolenic acid (C18:3) incubation. The reaction mixture consisted of 200 μM C18:3 incubated with 200 μL of protein solution and Tris-HCl buffer (0.1 M, pH 7.5) in a total volume of 500 μL. α-DOX products and the residual C18:3 were extracted with ethyl acetate; HDT was quantified by reverse-phase HPLC-UV after 2,4-dinitrophenylhydrazine derivatization, following the established procedure described in Kohlmann et al. [40].

### Analysis of secondary metabolites and phytohormones and 2-HOT

Nicotine was analyzed by HPLC as described previously (Keinanen et al., 2001) with the following modification of the extraction procedure: approximately 100 mg of frozen tissue was homogenized in 1 mL of extraction buffer using the FastPrep extraction system (Savant Instruments, NY, USA). Samples were homogenized in FastPrep tubes containing 900 mg of lysing matrix (BIO 101) by shaking at 6.0 m/s for 45 s.

To analyze phytohormones and 2-hydroxy-octadecatrienoic acid (2-HOT), 200 mg leaf samples were extracted with ethyl acetate containing 40 ng/ml D_6_-ABA, D_4_-SA and JA-^13^C_6_-Ile and 200 ng/ml of D_2_-JA according to Wu et al. [41]. A 10 μl aliquot of the resulting extracts was analyzed by reverse-phase HPLC coupled to tandem mass spectrometry (HPLC/ESI-MS/MS). Phytohormones were separated from extracts at a flow rate of 100 μL min^-1 ^on a Pursuit C8 column (3 μm, 150 × 2 mm; Varian) using a binary solvent system (A: 0.05% *v/v *formic acid in deionized water; B: 0.05% *v/v *formic acid in methanol) in gradient mode. Multiple reaction monitoring (MRM) was conducted on a 1200 L MS/MS system (Varian, Palo Alto, CA, USA), operated in negative ionization mode. Parent-ion/daughter-ion selections and collision energies were set as follows: 213/59 (D_2_-dihydro-JA, 12V), 209/59 (JA, 12V), 328/136 (^13^C_6_-JA-Ile, 19V), 322/130 (JA-Ile, 19V), 269/159 (D_6_-ABA, 9V), 263/153 (ABA, 9V), 141/97 (D4-SA, 15V), 137/93 (SA, 15V) and 293/191 (2-HOT). The area beneath the MRM product ion peak was determined for each analyte and internal standard (IS). The quantity of the analyte was calculated according to the formula: analyte product ion peak area × (IS concentration/IS product ion peak area). A calibration curve obtained by the extraction and analysis of a pool leaf sample spiked with increasing of amounts 2-HOT (Larodan Fine Chemicals, Sweden) was used for 2-HOT quantification.

For anthocyanin measurements 11, 15 and 19 days old seedlings of WT and two IR_*α-dox*_S lines were extracted according to Rabino and Mancinelli [42]. Approximately 30 seedlings (100 mg) were harvested, immediately frozen in liquid nitrogen and stored at -80°C for at least 20 minutes. After grinding 200 μl of 1% HCl/methanol solution were added and incubated overnight (15 h) at room temperature with constant vortexing. After centrifugation (5000 g, 5 min) the absorbance of the supernatant and a dilution series of cyanidin-5-O-glucoside at 530 nm and 657 nm was determined. The amount of anthocyanin was calculated according to the absorbance difference (530 nm - 657 nm).

### Statistics

Data were analyzed using the statistic program StatView^® ^5.0 (SAS Institute Inc., Cary, NC, USA). All data were checked graphically for normal distribution and for variance homogeneity using the *F-test *and transformed if required to meet these assumptions. The general level of significance applied was α = 0.05.

The effects of Na*α-dox *silencing on *α*-DOX activity, Na*α-dox1 *and Na*α-dox2 *transcript accumulation, and levels of ABA and JA in leaves were determined by comparing transformed lines against the WT *N. attenuata *by unpaired *t-tests*. Data for transcript accumulation were cube-root transformed for normality and variance homogeneity. Because no transformation was found to meet the requirements for parametric statistical analyses, levels of anthocyanins were compared between WT and IR_*α-dox*_S lines by Mann-Whitney *U-tests*.

The tissue-specific expression was compared between the different tissues by individual 1-factorial *ANOVAs *followed by *Fisher's PLSD *for Na*α-dox1 *and the Na*α-dox2 *gene. The transcript accumulation of Na*α-dox1 *and Na*α-dox2 *in each tissue was compared by unpaired *t-tests*. Data were cube root-transformed for normality and variance homogeneity.

The growth parameter and transcript accumulation of Na*α-dox1 *and Na*α-dox2 *for the plants silenced by VIGS were compared for statistically significant differences between transformants by 1-factorial *ANOVA *followed by *Fisher's PLSD *for individual comparisons. Growth data were box cox-transformed (λ = 1 for rosette diameters and λ = 2 for stem length) and transcript data were cube root-transformed for normality and variance homogeneity. The leaf levels of phytohormones of control and *M. sexta *induced VIGS plants were compared for statistically significant differences by 2-factorial *ANOVA *with the factors induction (2 groups: control, *M. sexta *feeding) and silencing (5 groups: VIGSev WT, VIGS*α-dox1 *WT, VIGS*α-dox2 *WT, VIGSev in IR_*α-dox*_M, VIGS*α-dox2 *in IR_*α-dox*_M) followed by *Fisher's PLSD*. Additionally, levels of JA, SA and ABA were analyzed by 1-factorial *ANOVAs *for constitutive and *M. sexta *induced plants separately. Data for JA and SA were log-transformed and data for ABA were cube root-transformed for normality and variance homogeneity. Because no transformation was found to meet parametric assumptions, levels of nicotine in VIGS plants were compared for significant differences between transformants by Mann-Whitney *U-tests*.

## Authors' contributions

AS carried out the screening of stably transformed lines, participated in the identification of the Na*α-dox2 *gene, VIGS experiments, the metabolite analyses, performed the statistical analysis and participated in drafting the manuscript. EG performed the transcriptional analysis, participated in the identification of the Na*α-dox2 *gene, VIGS experiments, metabolite analyses, carried out the sequence alignment and participated in drafting the manuscript. ITB conceived the study and participated in its design and coordination and helped to draft the manuscript. All authors read and approved the final manuscript.

## Supplementary Material

Additional file 1 **Alignment of Na*α-dox1 *and Na*α-dox2 *cDNA**. Sequences were aligned in Bioedit using the ClustalW algorithm. Regions used to design the inverted repeat silencing construct: The region of the Na*α-dox1 *gene used for the stable silencing construct is displayed in purple and shares a match of 24 nucleotides with the Na*α-dox2 *gene (containing one mismatch). The virus-induced gene silencing constructs are highlighted in blue for Na*α-dox1 *and in red for Na*α-dox2*. Click here for file

Additional file 2 **Phylogenetic relationship based on deduced amino acids sequences of plant α-DOXs**. The consensus neighbor-joining tree was constructed after 5000 iterations with the MEGA 3.1 software after sequence alignment using the ClustalW algorithm embedded into Bioedit. The GenBank accession numbers of the sequences are displayed onto the tree for the following plant species: *Arabidopsis thaliana *(At), *Capsicum annuum *(Ca), *Cicer arietinum *(Car), *Medicago truncatula *(Mt), *Nicotiana attenuata *(Na), *Nicotiana tabacum *(Nt), O*ryza sativa *(Os), *Physcomistrella patens *(Pp), *Pisum sativum *(Ps), *Populus trichocarpa *(Pt), *Ricinus communis *(Rc), *Solanum lycopersicon *(Sl), *Turnera subulata *(Tsu), *Turnera scabra *(Tsc), *Vitis vinifera *(Vv). Click here for file

Additional file 3 **Primer specificity, anthocyanin levels, and single insertions**. (**A**) Gel electrophoresis of gene specific PCR products on TAE gel after Ethidium bromide staining. Plasmids (50 ng) containing either the Na*α-dox1 *gene (2 left lanes) or a 355 bp fragment of Na*α-dox2 *(2 right lanes) were amplified with (**B**) 2 primer pairs, designed to specifically amplify a 144 bp fragment of either Na*α-dox1 *(P1) or Na*α-dox2 *(P2). The bands revealed that only primer pair P1 amplified the plasmid containing the Na*α-dox1 *gene and only primer pair P2 amplified the plasmid containing the Na*α-dox2 *fragment. (**B**) Anthocyanin levels (mean ± SE of 3 biological replicates) measured in 100 mg tissue of 11, 15, and 19 days old seedlings of WT and T_3 _homozygous *Nicotiana attenuata *plants transformed with an inverted repeat (IR) construct to silence Na*α-dox1 *(line 2). (**C**) Southern analysis of three independently transformed IR_α-dox _lines (S1, M1 & M2) showing single insertion. Genomic DNA (10 μg) from individual plants was digested with EcoR1 and blotted onto a nylon membrane. The blot was hybridized with a PCR fragment of the *hygromycin phosphotransferase II *gene, specific for the selective marker on the T-DNA. Click here for file

Additional file 4 **Co-silencing Naα*-dox1 *and Naα*-dox2 *results in increased constitutive ABA, JA and SA levels**. Mean ± SE (*n *= 4 biological replicates) levels of (**A**) ABA, (**B**) JA, and (**C**) SA in untreated rosette leaves of wild-type (WT) *Nicotiana attenuata *plants transformed with an inverted repeat (IR) construct to silence Na*α-dox1*. In contrast to lines silenced for Na*α-dox1*only (IR_*α-dox*_*M*), lines that were co-silenced for Na*α-dox1 *and Na*α-dox2 *have higher constitutive levels of ABA, JA, and SA. Asterisks signify significant differences between IR_*α-dox *_and WT plants (unpaired *t-test *for levels of ABA: M1 P = 0.65, M2 P = 0.30, S1 P = 0.001, S2 P = 0.03; JA: M1 P = 0.09, M2 P = 0.51, S1 P = 0.001, S2 P = 0.02, SA: M1: P = 0.43, M2: P = 0.36, S1 P = 0.05, S2 P = 0.002). Leaf SA levels (mean ± SE of plants that (**D**) had be fed on by *Manduca sexta *larvae for 4 days (*n *= 6) or (**E**) were undamaged (*n *= 5). WT and IR_*α-dox*_M plants were transiently silenced for Na*α-dox1 *and Na*α-dox2 *by virus-induced gene silencing (VIGS) or transformed with an empty vector (ev). Plants were inoculated with VIGS constructs when 22 days old and Larvae were applied on day 39. Though SA tended to be reduced in all *α*-*dox *silenced plants, this was only significant for VIGS *Na α-dox2 *(2-factorial ANOVA: for factor transformant F_4,35 _= 2.73, P = 0.04, for factor *M. sexta*: F_1,35 _= 0.018, P = 0.895; Fischer's PLSD: VIGS ev WT vs. VIGS *Na α-dox2 *WT P = 0.0037, VIGS ev WT vs. VIGS ev IR_*α-dox*_M P = 0.054). Click here for file

Additional file 5 **Table summarizing putative functions of *α-dox *genes in different plant species along the plant phylogenetic tree**. The occurrence of *α-dox *genes in different tissues or the regulation by pathogens, herbivores, wounding, or phytohormones it is indicated by "+" (usually a positive regulation except for ethylene in *Oryza sativa*), whereas the lack of expression is indicated by "-". References are given according to the reference list in the manuscript. Click here for file
