# Supplementary material for: The two α-dox genes of Nicotiana attenuata: overlapping but distinct functions in development and stress responses
Source: BMC Plant Biol. 2010 Aug 11;10:171. doi: 10.1186/1471-2229-10-171 (PMC3017789; doi:10.1186/1471-2229-10-171)
Supplement: Additional file 1 — Alignment of Naα-dox1 and Naα-dox2 cDNA. Sequences were aligned in Bioedit using the ClustalW algorithm. Regions used to design the inverted repeat silencing construct: The region of the Naα-dox1 gene used for the stable silencing construct is displayed in purple and shares a match of 24 nucleotides with the Naα-dox2 gene (containing one mismatch). The virus-induced gene silencing constructs are highlighted in blue for Naα-dox1 and in red for Naα-dox2. [file 1471-2229-10-171-S1.PDF]

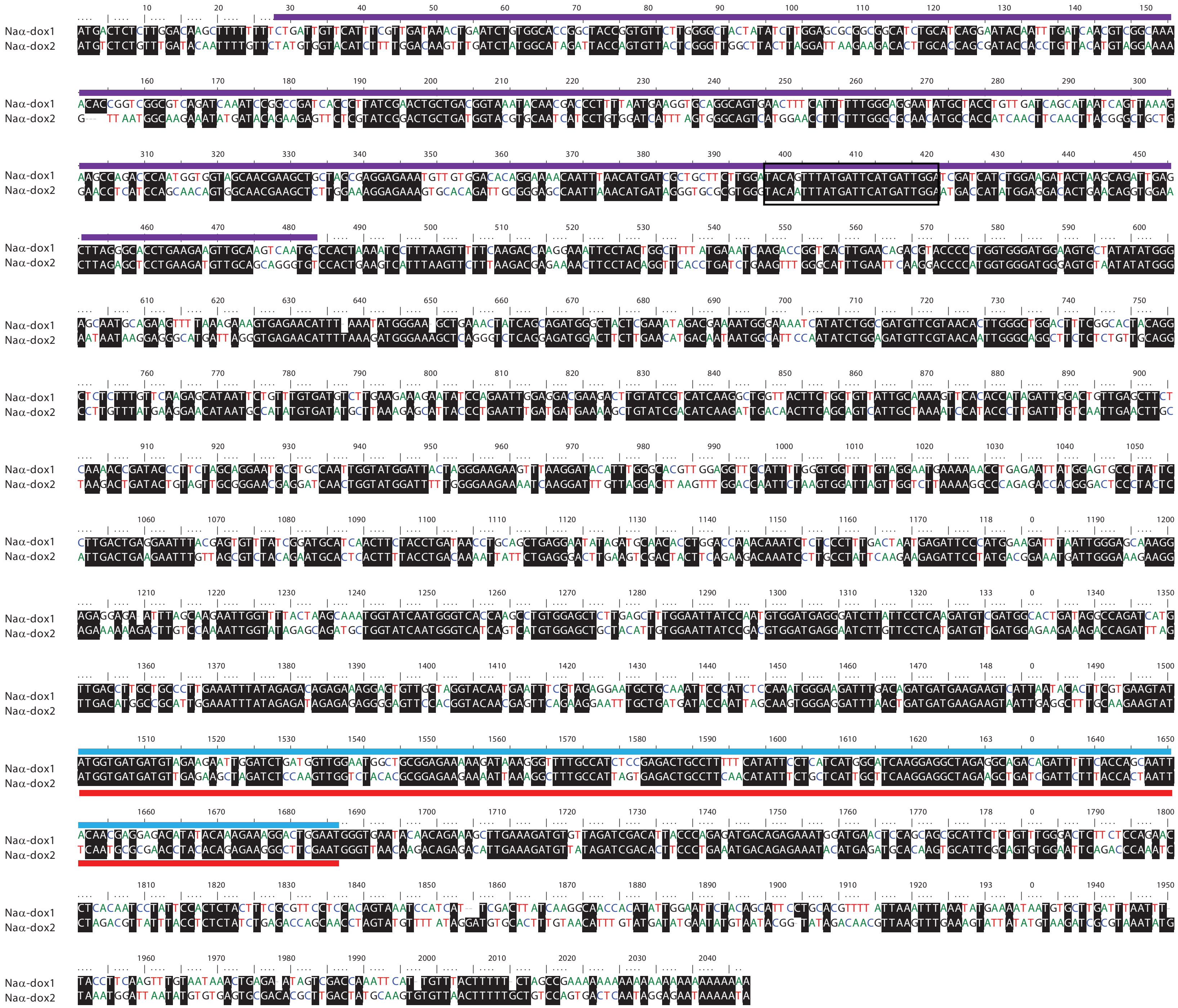
Naα-dox1
Naα-dox2
