# Supplementary figures and images for: The two α-dox genes of Nicotiana attenuata: overlapping but distinct functions in development and stress responses
Source: BMC Plant Biol. 2010 Aug 11;10:171. doi: 10.1186/1471-2229-10-171 (PMC3017789; doi:10.1186/1471-2229-10-171)

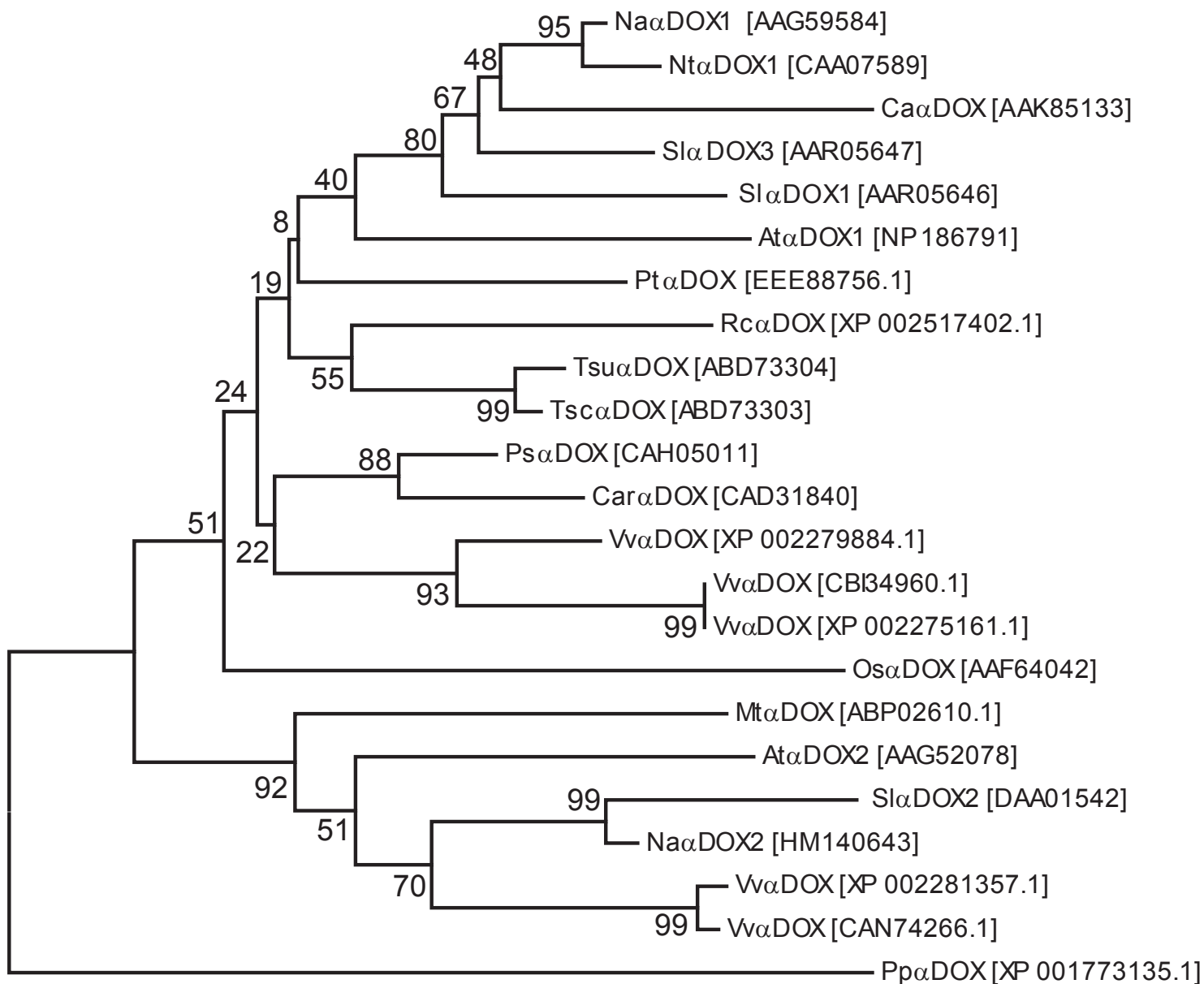

0.05

Supplement: Additional file 2 — Phylogenetic relationship based on deduced amino acids sequences of plant α-DOXs. The consensus neighbor-joining tree was constructed after 5000 iterations with the MEGA 3.1 software after sequence alignment using the ClustalW algorithm embedded into Bioedit. The GenBank accession numbers of the sequences are displayed onto the tree for the following plant species: Arabidopsis thaliana (At), Capsicum annuum (Ca), Cicer arietinum (Car), Medicago truncatula (Mt), Nicotiana attenuata (Na), Nicotiana tabacum (Nt), Oryza sativa (Os), Physcomistrella patens (Pp), Pisum sativum (Ps), Populus trichocarpa (Pt), Ricinus communis (Rc), Solanum lycopersicon (Sl), Turnera subulata (Tsu), Turnera scabra (Tsc), Vitis vinifera (Vv). [file 1471-2229-10-171-S2.PDF]

**A**

| Na $\alpha$ -dox1                                                                  |    | Na $\alpha$ -dox2 |    |
|------------------------------------------------------------------------------------|----|-------------------|----|
| P1                                                                                 | P2 | P1                | P2 |
| 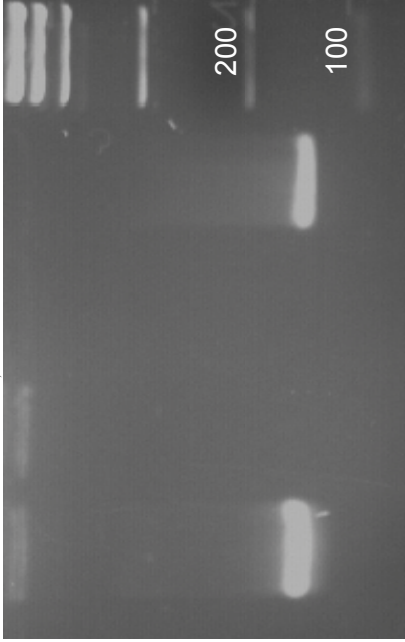 |    |                   |    |

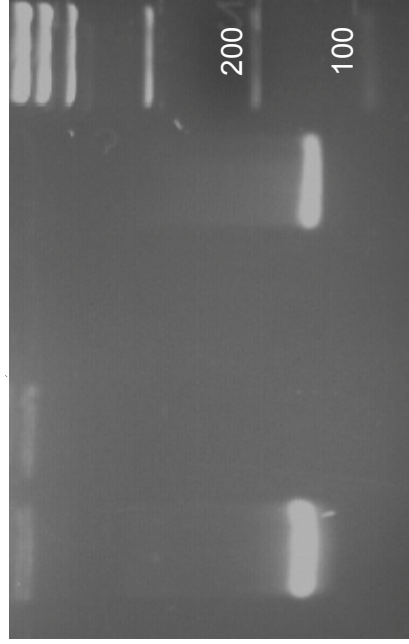**B**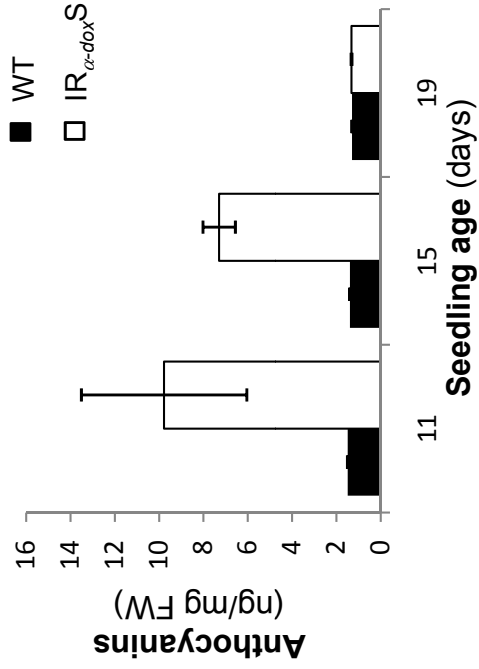**C**

| WT                                                                                | IR $\alpha$ -dox |    |    |    |  |  | pRESC | 5 $\mu$ PLOX |
|-----------------------------------------------------------------------------------|------------------|----|----|----|--|--|-------|--------------|
|                                                                                   | S1               | S2 | M1 | M2 |  |  |       |              |
| 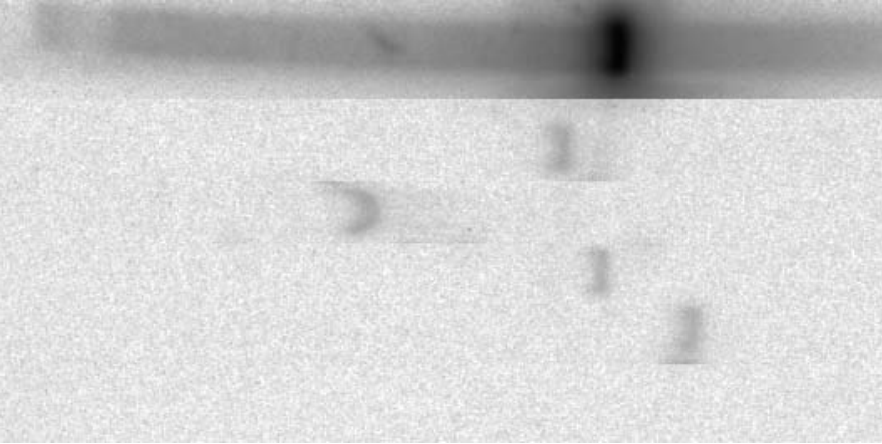 |                  |    |    |    |  |  |       |              |

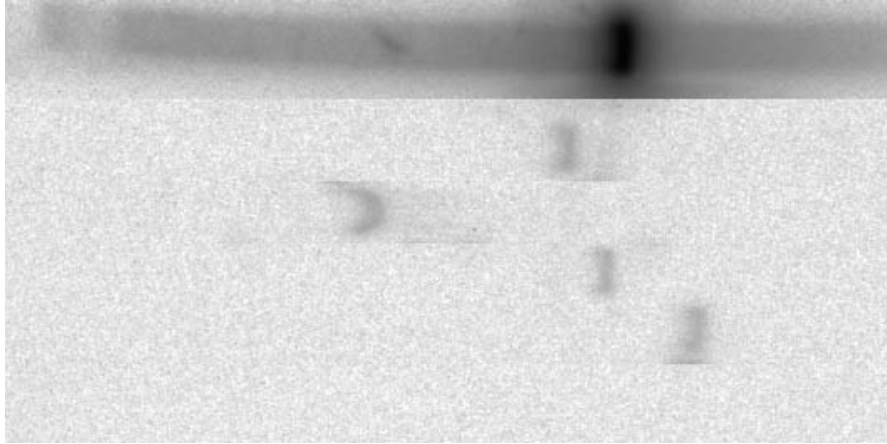

Supplement: Additional file 3 — Primer specificity, anthocyanin levels, and single insertions. (A) Gel electrophoresis of gene specific PCR products on TAE gel after Ethidium bromide staining. Plasmids (50 ng) containing either the Naα-dox1 gene (2 left lanes) or a 355 bp fragment of Naα-dox2 (2 right lanes) were amplified with (B) 2 primer pairs, designed to specifically amplify a 144 bp fragment of either Naα-dox1 (P1) or Naα-dox2 (P2). The bands revealed that only primer pair P1 amplified the plasmid containing the Naα-dox1 gene and only primer pair P2 amplified the plasmid containing the Naα-dox2 fragment. (B) Anthocyanin levels (mean ± SE of 3 biological replicates) measured in 100 mg tissue of 11, 15, and 19 days old seedlings of WT and T3 homozygous Nicotiana attenuata plants transformed with an inverted repeat (IR) construct to silence Naα-dox1 (line 2). (C) Southern analysis of three independently transformed IRα-dox lines (S1, M1 & M2) showing single insertion. Genomic DNA (10 μg) from individual plants was digested with EcoR1 and blotted onto a nylon membrane. The blot was hybridized with a PCR fragment of the hygromycin phosphotransferase II gene, specific for the selective marker on the T-DNA. [file 1471-2229-10-171-S3.PDF]

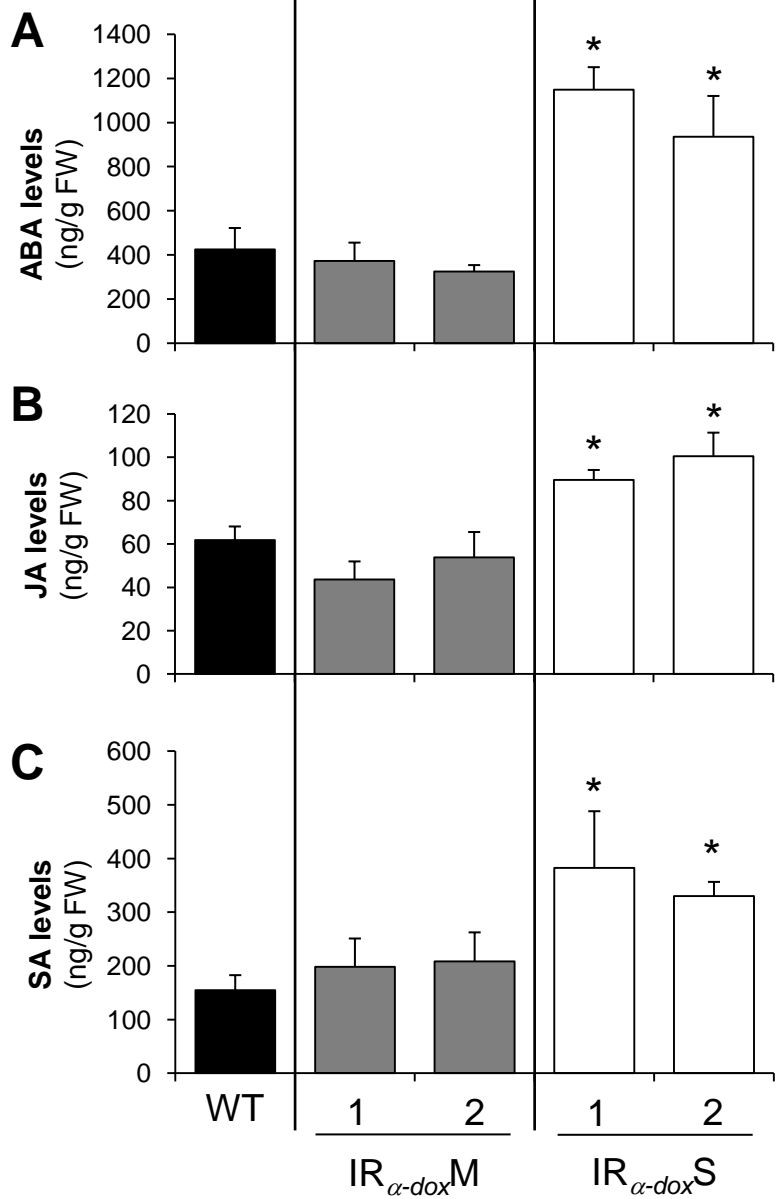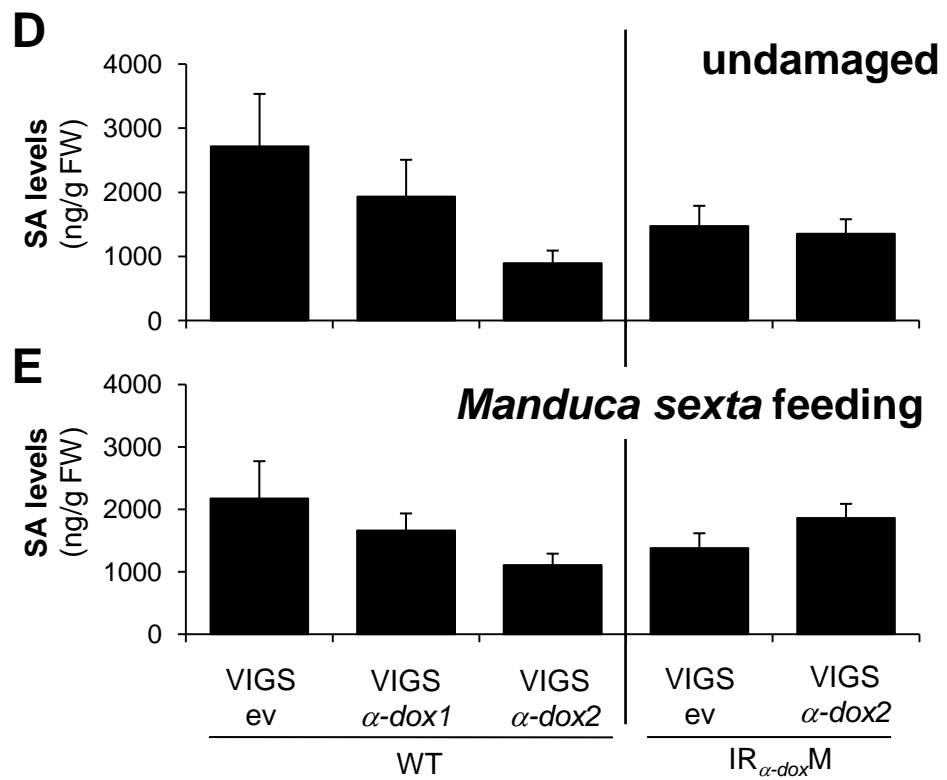

Supplement: Additional file 4 — Co-silencing Naα-dox1 and Naα-dox2 results in increased constitutive ABA, JA and SA levels. Mean ± SE (n = 4 biological replicates) levels of (A) ABA, (B) JA, and (C) SA in untreated rosette leaves of wild-type (WT) Nicotiana attenuata plants transformed with an inverted repeat (IR) construct to silence Naα-dox1. In contrast to lines silenced for Naα-dox1only (IRα-doxM), lines that were co-silenced for Naα-dox1 and Naα-dox2 have higher constitutive levels of ABA, JA, and SA. Asterisks signify significant differences between IRα-dox and WT plants (unpaired t-test for levels of ABA: M1 P = 0.65, M2 P = 0.30, S1 P = 0.001, S2 P = 0.03; JA: M1 P = 0.09, M2 P = 0.51, S1 P = 0.001, S2 P = 0.02, SA: M1: P = 0.43, M2: P = 0.36, S1 P = 0.05, S2 P = 0.002). Leaf SA levels (mean ± SE of plants that (D) had be fed on by Manduca sexta larvae for 4 days (n = 6) or (E) were undamaged (n = 5). WT and IRα-doxM plants were transiently silenced for Naα-dox1 and Naα-dox2 by virus-induced gene silencing (VIGS) or transformed with an empty vector (ev). Plants were inoculated with VIGS constructs when 22 days old and Larvae were applied on day 39. Though SA tended to be reduced in all α-dox silenced plants, this was only significant for VIGS Na α-dox2 (2-factorial ANOVA: for factor transformant F4,35 = 2.73, P = 0.04, for factor M. sexta: F1,35 = 0.018, P = 0.895; Fischer's PLSD: VIGS ev WT vs. VIGS Na α-dox2 WT P = 0.0037, VIGS ev WT vs. VIGS ev IRα-doxM P = 0.054). [file 1471-2229-10-171-S4.PDF]
