# Supplementary material for: The two α-dox genes of Nicotiana attenuata: overlapping but distinct functions in development and stress responses
Source: BMC Plant Biol. 2010 Aug 11;10:171. doi: 10.1186/1471-2229-10-171 (PMC3017789; doi:10.1186/1471-2229-10-171)
Supplement: Additional file 5 — Table summarizing putative functions of α-dox genes in different plant species along the plant phylogenetic tree. The occurrence of α-dox genes in different tissues or the regulation by pathogens, herbivores, wounding, or phytohormones it is indicated by "+" (usually a positive regulation except for ethylene in Oryza sativa), whereas the lack of expression is indicated by "-". References are given according to the reference list in the manuscript. [file 1471-2229-10-171-S5.PDF]

(core) eudicotyledons

Clade	Order	Family	Species	Gene	development: root	development: seedling leaves	development: mature leaves	development: detached leaves	development: flowers	elicited by: pathogens	elicited by: wounding	elicited by: herbivores	elicited by: SA	elicited by: JA	elicited by: ABA	elicited by: Ethylene	references
asterids	Solanales	Solanaceae	***Nicotiana attenuata***	*α-dox1*	–		+		–		+	+	–	+			[5, 30], this study
				α-dox2	+		+		+		–	–		–			
			Nicotiana tabacum	*α-dox1*			+			+	+		+	+			[9, 10, 11, 24]
				α-dox2						–	–		–	–			
			Solanum lycopersicum	*α-dox1*	+	–	–	–	–		+				+	+	[13, 28]
				α-dox2	–	+	–	–	+		+						
			Capsicum annuum	*α-dox1*						+	–		–	(+)		–	[17]
				α-dox2													
rosids	Brassicales	Brassicaceae	***Arabidopsis thaliana***	*α-dox1*	+	–	–	– (+)	+	+	+		+	–	–	–	[19, 28, 34]
				α-dox2	–	+	–	–	+								
	Fabales	Fabaceae	***Medicago truncatula***	*α-dox1*									+				[43][1]
				α-dox2													
			Pisum sativum	*α-dox1*	+	+											[23]
				α-dox2													
	Malpighiales	Passifloraceae	***Turnera scabra***	*α-dox1*					+								[35]
		Passifloraceae	***Turnera subulata***	*α-dox2*													
		Salicaceae	***Populus trichocarpa***	*α-dox1*													
				α-dox2													
		Euphorbiaceae	***Ricinus communis***	*α-dox1*													
				α-dox2													
	Vitales	Vitaceae	***Vitis vinifera***	*α-dox1*													
				α-dox2													
Liliopsida	Poales	Poaceae	***Oryza sativa***	*α-dox1*						+			–	+		+ *	[15]
				α-dox2													

* supression by ethylene

[43][1] Peleg-Grossman S, Golani Y, Kaye Y, Melamed-Book N, Levine A: **NPR1 protein regulates pathogenic and symbiotic interactions between Rhizobium and legumes and non-legumes.** 2009 *PLoS ONE* **4:** e8399
